# Desflurane improves lung collapse more than propofol during one-lung ventilation and reduces operation time in lobectomy by video-assisted thoracic surgery: a randomized controlled trial

**DOI:** 10.1186/s12871-022-01669-7

**Published:** 2022-04-29

**Authors:** Ryosuke Kawanishi, Nami Kakuta, Yoko Sakai, Yuki Hari, Hideto Sasaki, Ryo Sekiguchi, Katsuya Tanaka

**Affiliations:** 1 Division of Surgical Center, Tokushima University Hospital, 2-50-1 Kuramoto, Tokushima, 770-8503 Japan; 2 Department of Anesthesiology, Tokushima University Graduate School of Biomedical Sciences, Tokushima, Japan; 3 Department of Anesthesiology, Tokushima University Hospital, Tokushima, Japan

**Keywords:** Desflurane, Propofol, One-lung ventilation, Lung collapse

## Abstract

**Background:**

This study evaluated whether desflurane improved lung collapse during one-lung ventilation (OLV) more than propofol, and whether it could reduce the operation time of video-assisted thoracic surgery.

**Methods:**

Sixty patients undergoing lobectomy by video-assisted thoracic surgery (VATS) were randomly assigned to general anesthesia with desflurane or propofol. Lungs were inspected by thoracoscope at 10, 30, and 60 min after initiation of OLV. After surgery, the Lung Collapse Score, a composite of lung color and volume assessments, was assigned by two clinicians blinded to the anesthetic regimen. The primary outcome was operation time. The secondary outcome included the complication rate.

**Results:**

Of the 60 participants, 50 completed the study, 26 in Desflurane group and 24 in Propofol group. The Lung Collapse Scores at 30 and 60 min after OLV initiation were significantly better in Desflurane group than in Propofol group, and operation time was significantly shorter in Desflurane group (214 (57) min vs. 262 (72) min [mean (SD)], difference in means, -48; 95% CI, -85 to -11; *P* = 0.01). The incidence of multiple complications was 1/26 (3%) and 6/24 (25%) in Desflurane and Propofol group, respectively (relative risk, 0.1; 95% CI, 0.02 to 1.18; *P* = 0.04).

**Conclusions:**

Desflurane improved lung collapse during OLV and significantly shortened VATS lobectomy operation time compared to propofol in our studied patients. Desflurane resulted in fewer postoperative complications. Thus, desflurane may be an appropriate anesthetic during lobectomy by VATS requiring OLV.

**Trial registration:**

The study was registered with the University Hospital Medical Information Network (UMIN000009412). The date of disclosure of this study information is 27/11/2012. On this date, we registered the study into UMIN; patients were included from 2013 to 2014. However, on 11/27/2015, the UMIN system administrator suggested a detailed description. Thereafter, we added it to the Randomization Unit. Despite being prospective, it was retrospectively registered on UMIN for the above reasons.

## Background

One-lung ventilation (OLV) facilitates video-assisted thoracic surgery (VATS) in patients undergoing lobectomy [1, 2]. Because the intravenous anesthetic propofol does not inhibit hypoxic pulmonary vasoconstriction (HPV), it can contribute to oxygenation [3]. However, volatile anesthetic agents inhibit HPV [4, 5], suggesting that systemic oxygenation during OLV may be worse with volatile agents than with propofol. Benumof et al. reported that anesthesia with a minimum alveolar isoflurane concentration of 1.0 would inhibit the HPV response by approximately by 21%, but caused the shunt flow to increase by only 4% of the cardiac output [6]. Several clinical studies have reported no significant differences in systemic oxygenation between patients receiving propofol or volatile anesthesia during OLV [7–9].

Moreover, the choice of anesthetic may change the inflammatory responses in the lung, being lower in patients in whom anesthesia is maintained with volatile anesthetics than in those maintained with propofol during OLV [10–12]. A meta-analysis found that, compared with propofol, volatile anesthetic agents significantly reduced inflammatory responses of the lung and respiratory complications after thoracic surgery [13]. These findings suggested that volatile anesthesia may decrease postoperative complications by reducing the inflammatory response of the lungs during OLV.

Furthermore, volatile anesthetics may increase the risk of recurrence and death compared to intravenous anesthetics in cancer surgery. Administration of volatile anesthetic agents during cancer surgery reportedly suppresses the immune system and affect long-term survival compared with propofol [14]. However, the effects of volatile anesthetics and propofol on long-term survival after VATS lobectomy have not been compared.

We considered that desflurane may be superior to propofol from a completely different viewpoint. A spontaneous lung collapse on the operated side during thoracic surgery is important for good surgical exposure. As volatile anesthetics, including desflurane, have potent bronchodilatory effects [15], they may improve gas evacuation, resulting in good lung collapse during OLV, which would facilitate thoracic surgery. In the present study, we hypothesized that, compared to propofol, desflurane would enhance lung collapse during OLV and shorten operation time. This prospective, double-blind, randomized study tested this hypothesis by evaluating the effects of desflurane and propofol on lung collapse during OLV and the operation time of lobectomy by VATS. Additionally, we examined intraoperative oxygenation, postoperative complications, and long-term prognosis after VATS lobectomy.

## Materials and methods

The study protocol was approved by the local Ethics Committee of Tokushima University (Approval number: 1590), and written informed consent was obtained from all patients. The study was registered with the University Hospital Medical Information Network (UMIN000009412). No outside funding was received.

This study enrolled 60 patients scheduled to undergo lobectomy by VATS in Tokushima University Hospital from January 2013 to July 2014. To compare the operation time, the planned procedure was limited to VATS lobectomy. Our inclusion criteria were age 20‒75 years, American Society of Anesthesiologists physical status class 1‒2, Hugh‒Jones class 1‒2, and New York Heart Association class 1‒2. Our exclusion criteria were a history of cardiovascular or cerebrovascular disease and anticoagulant medication (because all study patients received epidural anesthesia), severe chronic obstructive pulmonary disease, defined as a percentage of forced expiratory volume in 1 s (%FEV_1.0_) < 50% of the predicted values; or severe restrictive lung disease, defined as a percentage of vital capacity (%VC) < 50% of the predicted values.

Patients were randomly assigned 1:1 to receive desflurane (Desflurane group) or propofol anesthesia (Propofol group) using a closed envelope method. Patients, surgeons, and the two clinicians who evaluated the Lung Collapse Score were blinded to the group assignment. Moreover, the desflurane vaporizer and syringe pump for propofol were covered with a cloth to blind the surgeons.

Anesthesiologists with > 5 years of experience and who were well-trained in anesthesia for thoracic surgery were in-charge, preventing delays in the operation time due to insufficient anesthesia techniques.

No patient was pre-medicated. An epidural catheter was inserted at the Th5‒8 level before general anesthesia induction. Anesthesia was induced in all patients by injection of 0.3 µg・kg^−1^・min^−1^ remifentanil, 1 mg/kg propofol, and 1 mg/kg rocuronium. General anesthesia was maintained with 4–6% desflurane and 0.2–0.3 µg・kg^−1^・min^−1^ remifentanil or with 2–4 µg/mL propofol (using target-controlled infusion systems) and 0.2–0.3 µg・kg^−1^・min^−1^ remifentanil, such that the BIS index was 40–60. Patients were intubated with a double-lumen endobronchial tube (DLT, Blue line®, Smith Medical US, Minneapolis, MN: 37 Fr for men and 35 Fr for women). The correct DLT position was confirmed by fiberoptic bronchoscopy.

After intubation, ventilators were set at an oxygen flow rate of 2 L/min. The fraction of inspired oxygen was 1.0; the tidal volume was 6 mL/kg, and the inspiratory/expiratory ratio was 1:1.5. The respiratory ratio was controlled such that the end-tidal carbon dioxide pressure was within 35‒45 mmHg, and the peak inspiratory pressure during ventilation was limited to 30 cmH_2_O. OLV began at the same time as surgery.

In both Desflurane and Propofol groups, dual-lung ventilation was continued before surgery, and OLV was continued after beginning with surgery, without interruption of ventilation. The surgical side of the DLT was clamped at OLV initiation and opened to the atmosphere during OLV to promote lung collapse. We did not perform a continuous suction technique on the surgical side unless specially requested by the surgeon.

The fraction of inspired oxygen was changed to 0.8 at 90 min after the start of surgery and to 0.6 at 120 min after the start of surgery. We did not use positive end-expiratory pressure during this study. Although we did not perform an alveolar recruitment maneuver, at the time of chest closure while checking with a thoracoscope, we took sufficient time to pressurize both lungs and confirm visually that the lungs were adequately inflated for all operations. After the operation was completed, X-rays were taken to confirm that both lungs were fully inflated, and the patient was extubated.

We injected 4 mg ephedrine when the systolic blood pressure decreased < 80 mmHg. Continuous epidural analgesia was started 60 min after the incision. Continuous epidural analgesia in all patients consisted of 5 mg/h levobupivacaine + 10 µg/h fentanyl. After surgery, all patients were admitted to the intensive care unit and monitored for approximately 24 h. Post-operatively, all patients were assessed daily for clinical signs of pulmonary complications until discharge. After hospital discharge, the patients were followed-up for approximately 5 years to evaluate cancer recurrence or death.

### Lung collapse score

**Fig. 1:**
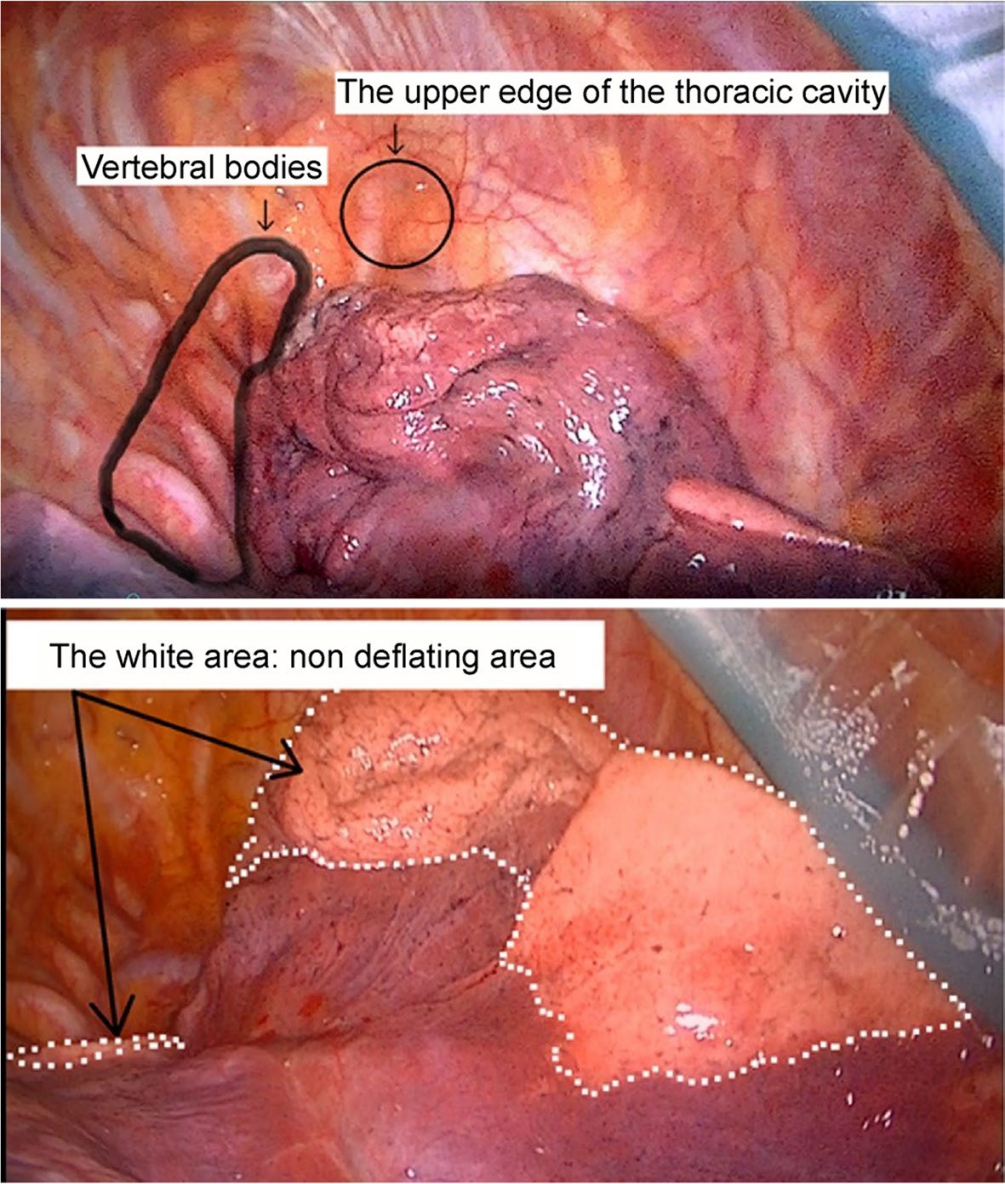
Assessment of Lung Collapse Score. The degree of lung collapse during one-lung ventilation was evaluated by measuring the Lung Collapse Score (Lung Collapse Score), evaluating the lung volume and the lung surface color. The upper panel shows a lung volume evaluation. In the distant view, with the video directed to the upper edge of the thoracic cavity, both the upper edge of the thoracic cavity and vertebral bodies are visible. The lung volume shown here is determined as 2. The lower panel shows a lung surface color evaluation. In this panel, as the white areas occupy between one-third and two-thirds of the lung surface, the color score is determined as 2

The degree of lung collapse during OLV was evaluated by measuring the Lung Collapse Score, which evaluates the lung volume (lung volume score) and lung surface color (lung color score, Fig. 1). In each patient, the operative lung was thoracoscopically inspected at 10, 30, and 60 min after OLV initiation and recorded on video. The Lung Collapse Score was assigned independently by two clinicians blinded to the anesthetic regimen using the recorded video at a later date. Lung volume scores were determined in the distant view with the thoracoscope directed to the upper edge of the thoracic cavity. If the upper edge of the thoracic cavity was not visible because of the lung, the lung volume score was 0. If the upper edge of the thoracic cavity was visible, but the vertebral bodies were not, the lung volume score was 1. If the upper edge of the thoracic cavity and vertebral bodies were visible, the lung volume score was 2. The lung color score was determined based on the size of the white areas, indicating a non-deflating area of the lung surface. If the white areas occupied more than two-thirds, between one-third and two-thirds, and less than one-third of the lung surface, the lung color scores were 1, 2, and 3, respectively. The Lung Collapse Score was defined as the sum of the lung color score and lung volume score. The average of the Lung Collapse Scores assigned by two clinicians was used for analysis.

### Outcomes

The primary outcomes were lung collapse and operation time. Operation time was defined as the time from the beginning of the operation, i.e., skin incision, to the end of wound closure. Secondary outcomes included oxygenation during OLV, and the number of pulmonary complications, such as post-extubation hypoxemia (a post-extubation P/F ratio < 200 mmHg), radiographically diagnosed pneumonia, and radiographically diagnosed atelectasis, radiographically diagnosed effusion, fistula, reintubation, systemic inflammatory response syndrome, surgical sight infection, surgical revision, and death. Inflammatory markers, such as white blood cell counts and C-reactive protein concentrations, were compared between Desflurane and Propofol groups. The 5-year postoperative recurrence-free survival rate and overall survival rate were compared as well between groups.

### Statistical analysis

All statistical analyses were performed using SPSS version 26 (IBM SPSS Corp., Chicago, IL, USA). Based on a previous study [16], the power calculation showed a mean (SD) duration of lobectomy by VATS of 192 (45) min. Calculations indicated that to show a 40 min reduction in operation time would require 21 patients per group, using a two-sided design and a significance level of 5% (α = 0.05), with a probability of 80% (β = 0.20). Allowing for potential dropouts, 30 patients per group were enrolled. A histogram and the Shapiro–Wilk test were used to assess data distribution. Continuous data are reported as mean (SD). Categorical data are reported as counts and percentages. The two groups were compared using Student's *t*-tests for continuous data or Fisher's exact tests for categorical data. The Chi-square test was used to compare pathological stages. A linear regression analysis was performed to examine the effect of specific factors on operation time and Lung Collapse Score. We also conducted a multiple regression analysis to identify the factors affecting the operation time among the eight factors of patient background, LCS60, and blood loss. The 5-year postoperative recurrence-free survival rate and overall survival rate were assessed using the Kaplan–Meier method and were compared by log-rank tests. *P* < 0.05 was considered statistically significant.

## Results

**Fig. 2:**
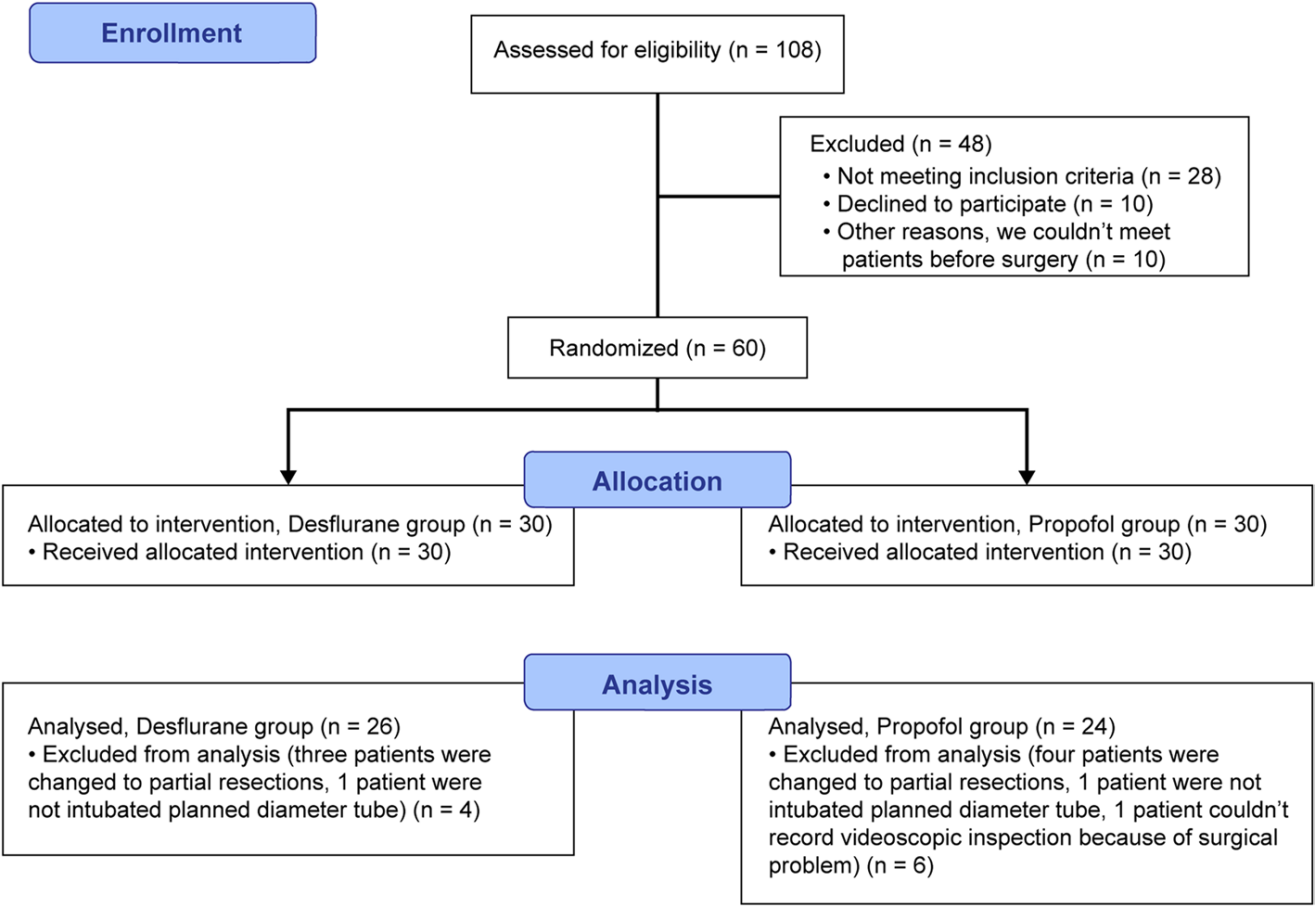
Flow diagram of this study

Of the 60 patients enrolled, 10 were excluded. Eight patients were excluded because their operations were converted to partial resection. Two patients were excluded because a DLT with the planned inner diameter could not be intubated. Of the 50 remaining participants, 26 were in Desflurane group and 24 in Propofol group (Fig. 2). One patient in each group was used the continuous suction technique at the surgeon's request.

Patient characteristics are shown in Table 1. Despite the randomization, there were significant differences in patient background between groups regarding age and %VC. We have three thoracic surgery teams at our institution, with average VATS lobectomy times of 254 (84), 228 (56), and 217 (53) min, respectively, in this study period. There was no significant difference in the variability of the distribution of the three teams between the two groups (Table 1). The pathological cancer stage did not differ significantly between groups (Table 2).

**Table 1:** Demographic and clinical characteristics of patients in the Desflurane and Propofol groups

	Desflurane group	Propofol group	Relative risk (95% CI)	*P* value
	*N* = 26	*N* = 24		
Female, n/total N (%)	12/26 (46%)	11/24 (45%)	1.00 (0.55 to 1.83)	1.00^a^
ASA-PS, 1/total N (%)	7/26 (26%)	5/24 (20%)	1.29 (0.47 to 3.52)	0.74^a^
Resection side, L/total N (%)	9/26 (34%)	11/24 (45%)	0.75 (0.38 to 1.49)	0.56^a^
Right Upper Lobe, L/total N (%)	9/26 (34%)	7/24 (29%)		0.88^a^
Right Middle Lobe, L/total N (%)	3/26 (11%)	2/24 (8%)		
Right Lower Lobe, L/total N (%)	5/26 (19%)	4/24 (16%)		
Left Upper Lobe, L/total N (%)	7/26 (26%)	7/24 (29%)		
Left Lower Lobe, L/total N (%)	2/26 (7%)	4/24 (16%)		
Thoracic surgery team,	12 (46%) / 10 (38%) /	8 (33%) / 14 (58%) /		0.35^a^
team A / team B / team C	4 (15%)	2 (8%)		
COPD, n/total N (%)	2/26 (7%)	5/24 (20%)	0.36 (0.07 to 1.72)	0.23^a^
Asthma, n/total N (%)	0/26 (0%)	2/24 (8%)	N/A	0.22^a^
			*The difference in Means (95% CI)*	
Age (yr), mean (SD)	61.9 (6.9)	66.0 (5.6)	-4.1 (-7.7 to -0.5)	0.02^b^
Height (cm), mean (SD)	159 (5.8)	159 (7.6)	0.2 (-3.5 to 4,1)	0.89^b^
Weight (kg), mean (SD)	57.5 (7.6)	60.7 (12.1)	-3.1 (-8.8 to 2.5)	0.26^b^
%VC (%), mean (SD)	119 (13.7)	110 (12.3)	9.4 (2.0 to 16.9)	0.01^b^
%FEV_1.0_ (%), mean (SD)	76.1 (5.5)	75.8 (10.0)	0.3 (-4.2 to 4.8)	0.89^b^

Relative risks are for Desflurane group relative to Propofol group; the difference is (Desflurane group – Propofol group)

Average VATS lobectomy times in this study period of team A: 254 (84), team B: 228 (56), and team C: 217 (53) min, respectively

*VC* vital capacity, *FEV* forced expiratory volume, *COPD* chronic obstructive pulmonary disease, *L* left, *R* right, *ASA-PS* American Society of Anesthesiologists physical status, *CI* confidence interval, *SD* standard devision

^a^Fisher's exact test

^b^Student's *t*-test

**Table 2:** Pathological cancer stage in Desflurane and Propofol groups

	Stage IA	Stage IB	Stage II A	Stage III A	Others (e.g., metastasis)
Desflurane group, n/total N (%) (*N* = 26)	12/26 (46%)	4/26 (15%)	3/26 (11%)	4/26 (15%)	3/26 (11%)
Propofol group, n/total N (%) (*N* = 24)	12/24 (50%)	3/24 (12%)	1/24 (4%)	4/24 (16%)	4/24 (16%)

Chi-square test showed *P* = 0.87

The Lung Collapse Score was measured 50 times each at 10, 30, and 60 min, the total number of repitition was 150 times. Of these, the number of times the scores were in perfect agreement was 90 times or 60%; the number of times the scores deviated by 1 point was 58 times or 39%, and the number of times the scores deviated by 2 points was twice or 1%.

Although the Lung Collapse Score did not differ significantly between groups at 10 min after OLV initiation, the Lung Collapse Score at 30 min (Lung Collapse Score30: LCS30) and 60 min (Lung Collapse Score60: LCS60) were significantly higher in Desflurane group than in Propofol group. Moreover, the mean (SD) operation time was 214 (57) min and 262 (72) min in Desflurane and Propofol groups, respectively (difference in means, -48; 95% CI, -85 to -11; *P* = 0.01). To examine the difference of operation time more carefully, we also compared the operation time of two groups except for cases with exceptionally long operation times. Based on the histogram analysis, two patients in Propofol group were determined to be outliers in terms of operation time (operation time: 469, 394 min). When excluding these two patients, the operation time was significantly shorter (DES vs. PROPO: 214.5 (57.6) min vs. 247.3 (51.5) min; *P* = 0.04), bleeding was significantly lower (DES vs. PROPO: 59.3 (73.4) mL vs. 138.6 (164.5) mL; *P* = 0.04), and the lung collapse score at 60 min was significantly higher (DES vs. PROPO: 4.5 (0.5) vs. 4.0 (0.6); *P* = 0.01) in Desflurane group than in Propofol group.

At 60 min from the start of surgery, the surgeon was asked to rate his satisfaction with the lung collapse on a 10-point scale. There was no significant difference in satisfaction with lung collapse between the groups (DES vs. PROPO: 9.5 (0.6) vs. 9.4 (0.8), repsectively; *P* = 0.46).

Secondary outcomes, including PaO_2_ during OLV, duration of hospital stay, and concentrations of systemic inflammatory mediators, did not differ significantly between the groups (Table 3).

**Table 3:** Intraoperative Lung Collapse Score and other outcomes

	Desflurane group	Propofol group	Difference in Means	*P*-value
	*N* = 26	*N* = 24	(95% CI)	
Lung Collapse Score (LCS)				
LCS10: 10 min after the initiation of OLV, mean (SD)	2.0 (0.4)	1.9 (0.5)	0.0 (-0.2 to 0.2)	0.85
LCS30: 30 min after the initiation of OLV, mean (SD)	3.4 (0.6)	3.0 (0.6)	0.3 (0.0 to 0.6)	0.03
LCS60: 60 min after the initiation of OLV, mean (SD)	4.5 (0.6)	4.1 (0.6)	0.4 (0.1 to 0.7)	0.00
Operation time (min), mean (SD)	215 (58)	263 (72)	-48 (-85 to -11)	0.01
Amount of bleeding (mL), mean (SD)	59 (73)	135 (159)	-75 (-148 to -6)	0.04
PaO_2_ (mmHg)				
Before OLV, mean (SD)	492 (52)	457 (82)	35 (-3 to 74)	0.07
10 min after initiation of OLV, mean (SD)	227 (87)	194 (116)	33 (-25 to 91)	0.25
30 min after initiation of OLV, mean (SD)	216 (81)	171 (110)	45 (-9 to 100)	0.1
60 min after initiation of OLV, mean (SD)	183 (72)	154 (68)	29 (-11 to 69)	0.15
White blood cells (count × 10^3^/μl)				
Before surgery, mean (SD)	5.2 (1.5)	6.1 (2.0)	-0.8 (-1.8 to 0.1)	0.08
Postoperative day 1, mean (SD)	10.3 (3.5)	10.2 (3.4)	0.0 (-1.9 to 2.0)	0.94
Postoperative day 3, mean (SD)	9.1 (2.3)	9.0 (1.8)	0.1 (-0.9 to 1.3)	0.74
C-reactive protein (mg/dl)				
Before surgery, mean (SD)	0.1 (0.1)	0.2 (0.4)	-0.1 (-0.2 to 0.0)	0.25
Postoperative day 1, mean (SD), mean (SD)	3.8 (1.7)	4.0 (1.7)	-0.1 (-1,1 to 0.7)	0.69
Postoperative day 3, mean (SD)	4.3 (3.3)	5.7 (4.5)	-1.3 (-3.5 to 0.8)	0.23

Differences are (Desflurane group – Propofol group)

Student's *t-*test was used for all tests

*LCS* Lung Collapse Score, *OLV* one-lung ventilation, *CI* confidence interval, *SD* standard devision

**Fig. 3:**
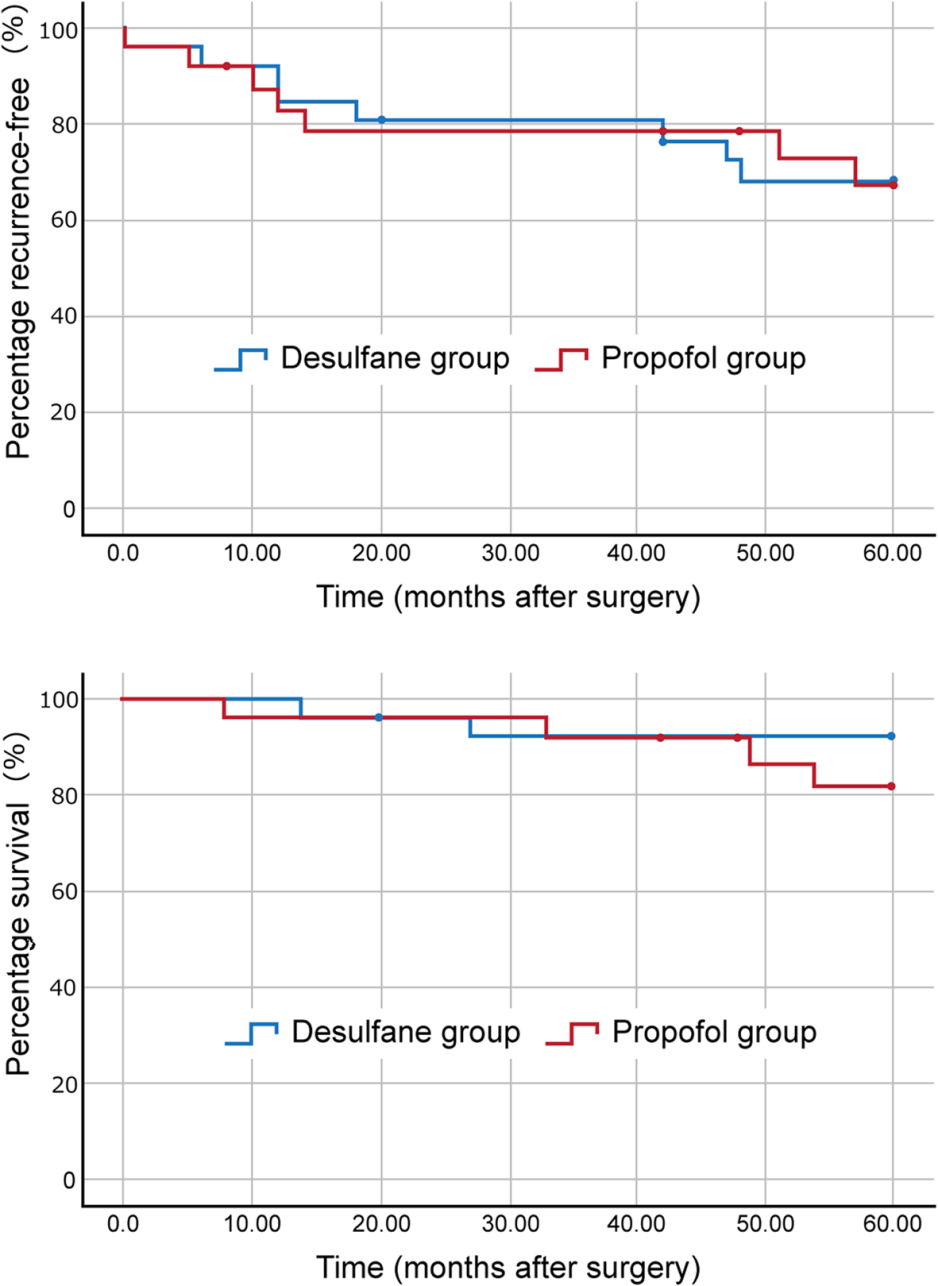
Five-year recurrence-free survival rate or overall survival rate after lobectomy under desflurane and propofol anesthesia. The recurrence-free survival rate and survival rates did not differ in these two groups (recurrence-free survival rate: *P* = 0.98, survival rate: *P* = 0.33)

Postoperative adverse events are shown in Table 4. The incidence of post-extubation hypoxemia was 0/26 (0%) in Desflurane group and 4/24 (16%) in Propofol group (relative risk was not available; *P* = 0.04). The incidence of multiple complications was 1/26 (3%) in Desflurane group and 6/24 (25%) in Propofol group (relative risk, 0.1; 95% CI, 0.02 to 1.18; *P* = 0.04). Six patients died during the postoperative follow-up period. The mean follow-up period for non-deceased patients was 57.4 weeks. The 5-year postoperative recurrence-free survival rate and overall survival rate did not differ between these groups (Fig. 3).

**Table 4:** Postoperative outcomes

Outcomes	Desflurane group	Propofol group	Relative risk (95% CI)	*P* value
	*N* = 26	*N* = 24		
*Adverse event*				
Pneumonia, n/total N (%)	1/26 (3%)	2/24 (8%)	0.46 (0.04 to 4.77)	0.60^a^
Atelectasis, n/total N (%)	4/26 (15%)	5/24 (20%)	0.73 (0.22 to 2.43)	0.72^a^
Surgical revision, n/total N (%)	0/26 (0%)	2/24 (8%)	N/A	0.22^a^
Fistula, n/total N (%)	1/26 (3%)	1/24 (4%)	0.92 (0.06 to 13.95)	1.00^a^
Effusion, n/total N (%)	1/26 (3%)	2/24 (8%)	0.46 (0.04 to 4.77)	0.60^a^
Surgical sight infection, n/total N (%)	1/26 (3%)	3/24 (12%)	0.30 (0.03 to 2.76)	0.34^a^
Reintubation, n/total N (%)	0/26 (0%)	2/24 (8%)	N/A	0.22^a^
SIRS, n/total N (%)	1/26 (3%)	5/24 (20%)	0.18 (0.02 to 1.46)	0.09^a^
Post-extubation hypoxemia, n/total N (%)	0/26 (0%)	4/24 (16%)	N/A	0.04^a^
Death, n/total N (%)	0/26 (0%)	0/24 (0%)	N/A	N/A
Total, n/total N (%)	9	26		
Multiple adverse events, n/total N (%)	1/26 (3%)	6/24 (25%)	0.15 (0.02 to 1.18)	0.04^a^
5-year postoperative recurrence, n/total N (%)	8/26 (30%)	7/24 (29%)	1.05 (0.45 to 2.46)	1.00^a^
5-year postoperative death, n/total N (%)	2/26 (7%)	4/24 (16%)	0.46 (0.09 to 2.29)	0.40^a^
			*The difference in Means (95% CI)*	
Hospital stay (days), mean (SD)	13 (14)	18 (22)	-5.5 (-16.3 to 5.2)	0.30^b^
Postoperative recurrence-free period (months), mean (SD)	48.5 (3.8)	48.2 (4.3)	N/A	0.98^c^
Postoperative survival period (months), mean (SD)	56.9 (2.1)	55.8 (2.3)	N/A	0.33^c^

Relative risks are for Desflurane group relative to Propofol group; differences are (Desflurane group – Propofol group)

*SIRS* Systemic inflammatory response syndrome, *CI* confidence interval, *SD* standard devision, *N/A* Not available

^a^Fisher's exact test

^b^Student's *t*-test

^c^*P-*value from log-rank test

Linear regression analysis showed no significant association between operation time and age or between operation time and %VC. There was no significant association of operation time with height, body weight, body mass index, and %FEV_1.0_. In contrast, LCS30 and LCS60 were related to operation time (LCS30: *R* = -0.30, *P* = 0.04, LCS60: R = -0.31, *P* = 0.03; Table 5). A weak positive correlation was found between %VC and Lung Collapse Score10. However, no other significant correlation was found between Lung Collapse Score 10/30/ 60 and age, or between Lung Collapse Score 10/30/60 and %VC (Table 6).

**Table 5:** Pearson's correlation of factors with operation time

	Pearson's correlation coefficients	*P* value
Age	0.24	0.09
Height	0.27	0.06
Bodyweight	0.17	0.23
BMI	0.08	0.60
%VC	-0.08	0.57
%FEV_1.0_	-0.08	0.60
Lung Collapse Score10	-0.06	0.66
Lung Collapse Score30	-0.3	0.04
Lung Collapse Score60	-0.31	0.03
Bleeding	0.35	0.01

*BMI* body mass index, *VC* vital capacity, *FEV* forced expiratory volume

**Table 6:** Pearson's correlation between two factors

	Pearson's correlation coefficients	*P*-value
*Between Lung Collapse Score10*		
Age	0.30	0.02
%VC	-0.20	0.14
*Between Lung Collapse Score30*		
Age	0.04	0.77
%VC	0.16	0.24
*Between Lung Collapse Score60*		
Age	-0.11	0.41
%VC	0.21	0.13

*LCS* Lung Collapse Score, *VC* vital capacity

A multi-regression analysis revealed that the factors that had a statistically significant effect on operation time were height (standardized coefficients Beta: 0.33, *P* = 0.01), LCS60 (standardized coefficients Beta: -0.31, *P* = 0.02), and bleeding (standardized coefficients Beta: 0.27, *P* = 0.01) at the 0.05 level, (adjusted R^2^: 0.241, *P*-value of prediction equation: 0.001; Table 7).

**Table 7:** Coefficients of explanatory variables determined significant by multiple regression analysis (*N* = 50)

Variables	Standardized Coefficients Beta	Sig
Height	0.33	0.01
LCS60	-0.31	0.02
Bleeding	0.27	0.03

Objective variavles: operation time, *P* < 0.05, *R*^2^ = 0.28, *P* = 0.001

## Discussion

This prospective randomized study showed that desflurane anesthesia improved lung collapse during OLV and, consequently, shortened the operation time of lobectomy by VATS, and reducing postoperative adverse events. Improvement of lung collapse during OLV might have improved the field of view over the surgical site of the lobectomy, resulting in a shorter operation time. These beneficial effects may help to reduce postoperative adverse events.

The mechanism by which desflurane promotes spontaneous lung collapse remains unclear. Lung collapse is affected by different factors [17]. In the early phase, oxygen in the alveoli outflows mainly via the respiratory tract. In the later phase, after the peripheral respiratory tract is obstructed by the initial oxygen outflow, oxygen in the alveoli diffuses and is taken up into the pulmonary arteries. Desflurane may promote oxygen egress from the peripheral respiratory tract and oxygen diffusion into the pulmonary arteries. Bronchodilation by desflurane may improve oxygen drainage via the peripheral respiratory tract during OLV.

Moreover, desflurane inhibition of HPV may improve oxygen diffusion into the pulmonary arteries during OLV by not reducing blood flow to the pulmonary artery on the surgical side. Moreover, as desflurane has higher specific gravity than oxygen (desflurane specific gravity: 1.47, oxygen specific gravity: 1.10) [18], alveolar gas may have higher specific gravity than propofol. This difference in the specific gravity of alveolar gas may accelerate alveolar gas evacuation via the peripheral respiratory tract during lung collapse in desflurane anesthesia compared to propofol anesthesia. Bronchodilation, HPV suppression, and specific gravity differences would each have produced only a minimal effect. However, together, these effects might have resulted in significantly superior lung collapse in Desflurane group.

Interestingly, the difference in lung collapse progression became more apparent over time, even though the operative lung was not ventilated during OLV. Absorptive atelectasis is considered a significant factor for lung collapse [19]. Blood from the pulmonary artery continuously flows in the operative lung during OLV. Compared with desflurane, propofol does not inhibit HPV. Therefore, Propofol group was expected to exhibit decreased blood flow to the operative lung compared to that in Desflurane group. To achieve complete removal of alveolar gas, it is important to induce alveolar gas absorption into the pulmonary blood flow, i.e., absorptive atelectasis. However, in Propofol group, induction of absorptive atelectasis may be delayed due to an HPV-induced decrease in pulmonary blood flow on the operative side. Therefore, the lung collapse in Propofol group may have delayed over time compared to Desflurane group, even when the operative lung was not ventilated.

Studies comparing sevoflurane and desflurane suggest that the two anesthetics have similar bronchodilatory and HPV-inhibitory effects [15, 20]. Here, the bronchodilatory and HPV-inhibitory effects of desflurane are assumed to explain its superiority to propofol for lung collapse. Therefore, sevoflurane may have a similar effect in promoting lung collapse.

In the present study, we evaluated the quality of lung collapse on the operative side during OLV. There have been no reports of objective measurements of the quality of lung collapse in clinical studies, although one study reported subjective measurements [21]. Two animal studies reported objective measurements of lung collapse using an invasive method not clinically practicable [22, 23].

The Lung Collapse Score we used is a novel method for assessing lung collapse of unknown reliability. However, with a perfect agreement rate of 60% and a discrepancy ≧2 points in the 1% range between two clinicians, we believe that we were able to maintain a sufficient level of agreement for an evaluation method based on appearance. Additionally, we showed significant relationships of both the LCS30 and LCS60 with operation time. These findings suggest the usefulness of the Lung Collapse Score for assessing the quality of lung collapse.

We could not measure pulmonary artery pressure and only assessed the trend of intraoperative oxygenation. There was no significant improvement in oxygenation between 30 and 60 min after OLV initiation in any groups (Table 3). In our study model, we could not find a second HPV peak 40–45 min after OLV initiation [24].

### Operation time of lobectomy by VATS

This research is the first to show a difference in operation time between the two groups. VATS procedures for lung cancer include lobectomy, segmentectomy, and partial resection in our institute. Lobectomy requires ≧3 vascular treatments (≧2 pulmonary arteries and ≧1 pulmonary vein), whereas segmentectomy requires two vascular treatments (one pulmonary artery and one pulmonary vein), and partial resections do not require any vascular treatments. Vascular treatments require careful attention, and are considered the bottleneck determining the operation time. Therefore, the operation time for lobectomy is longer than for segmentectomy and partial resection. Since the primary outcome of this study was operation time, we limited procedures to VATS lobectomy. Previous reports included both lobectomy and others, or thoracotomy and VATS [10, 25–27], which may explain why their results differed from ours.

Moreover, many of these reports tend to report a shorter operation time in the inhalational anesthetic group. Conno's report showed significantly shorter OLV duration with inhalational anesthetics [25]. This outcome can be considered similar to our results.

In our study, Desflurane group exhibited better lung collapse than that in Propofol group. We also found a negative correlation between LCS and operative time. These results suggest a better lung collapse in Desflurane group, which facilitated obtaining a good surgical view quickly, leading to a faster and safer surgical procedure, thus shortening the operation time.

### Intraoperative oxygenation

In a study by Cho et al. that examined the effects of desflurane and propofol on oxygenation during OLV, desflurane significantly worsened oxygenation during OLV compared to propofol [28]. Conversely, there was no significant difference in oxygenation during OLV between groups in the present study. The desflurane concentration in their study (5‒7%) was higher than that in ours (4‒6%). Since desflurane enhances HPV suppression in a concentration-dependent manner, differences in desflurane concentration may have affected the results.

### Postoperative complications

We demonstrated that the number of patients with post-extubation hypoxia and multiple adverse events was significantly smaller in Desflurane group than in Propofol group. Recently, several studies demonstrated that administration of volatile anesthetics during lung resection surgery reduces the frequency of postoperative pulmonary complications, as volatile anesthetics attenuate the pulmonary and systemic inflammatory responses [13, 25, 29]. Molecular studies on the targets of volatile anesthetics on neutrophils, monocytes, and macrophages reveal that volatile anesthetics significantly reduce neutrophil recruitment and phagocytosis by targeting multiple molecules [30, 31]. Compared with propofol, volatile anesthetics reduce local inflammatory responses during OLV [25–27, 29]. However, no previous study suggested that volatile anesthetics are superior to intravenous anesthetics in reducing postoperative complications after lobectomy by VATS from these viewpoints.

### Long-term recurrence and survival rates

The use of volatile anesthetics may result in more frequent small metastases after cancer surgery and affect long-term recurrence and survival rates [32]. However, a previous study showed that desflurane did not reduce the number of short- and long-term major complications after standard lung surgery compared to propofol anesthesia [33]. The present study similarly showed that desflurane anesthesia did not worsen the 5-year postoperative recurrence-free survival rate or overall survival rate compared with propofol anesthesia. Despite our small sample size, we could confirm that desflurane did not worsen the cancer prognosis in our patients.

### Limitations

This study had several limitations. Despite the randomization, there was a significant difference in %VC and age between groups, but neither factor was significantly associated with operation time. The effects of differences in the patient background on operation time and lung collapse were considered irrelevant. Because we conducted the statistical analyses after collecting the cases, it was impossible to eliminate the significant differences in patients’ backgrounds. Thus, we conducted a multiple regression analysis to identify the factors affecting operation time among the eight factors of patient background, LCS60, and blood loss. It revealed that height, LCS60, and bleeding significantly affected the operation time. Based on the statistical analyses, we considered a minimal influence of %VC and age on the operation time (Table 8). Of these three factors, the amount of bleeding was rather considered a surgical outcome, as well as the operation time. This result also suggests that the Lung Collapse Score was an important factor affecting operation time. Promoting lung collapse provides the surgeon with a better surgical view, leading to a faster and safer operation. Resultantly, Desflurane group, which had a better lung collapse, had shorter surgery time and less bleeding.

**Table 8:** Coefficients when the removed variables are input (*N* = 50)

Variables	Standardized Coefficients Beta	Sig
Desflurane or Propofol group	0.19	0.16
Age	0.14	0.26
Male or female	0.13	0.41
Weight	-0.10	0.51
BMI	-0.06	0.60
FEV_1.0%_	0.04	0.76
%VC	-0.00	0.97

Another limitation is that Desflurane group also received 1 mg of propofol per body weight for anesthesia induction. Since general anesthesia induction with desflurane alone is difficult, we had to use propofol for induction. At our institute, the time from anesthesia induction to the beginning of surgery is approximately 60 min. Considering the metabolic speed of propofol, we considered the propofol concentration in Desflurane group to be negligibly low at OLV initiation.

Other limitations include the small sample size and the inclusion of patients from a single center. Large and prospective multicenter trials are needed to compare the duration of lobectomy by VATS in patients administered propofol or desflurane anesthesia.

## Conclusion

In conclusion, compared with propofol, desflurane improved lung collapse during OLV and significantly shortened the operation time of VATS lobectomy in our study patients. Additionally, desflurane reduced the postoperative complication rate. These results suggest that the good quality lung collapse induced by desflurane may enhance surgical exposure during VATS lobectomy, reducing operation time and postoperative adverse events.

## Data Availability

The datasets used or analyzed during the current study are available from the corresponding author on reasonable request.

## Abbreviations

VATS: Video-Assisted Thoracic Surgery
OLV: One-Lung Ventilation
UMIN: University Hospital Medical Information Network
HPV: Hypoxic Pulmonary Vasoconstriction
FEV: Forced Expiratory Volume
VC: Vital Capacity
SD: Standard Deviation
LCS60: Lung Collapse Score60
LCS30: Lung Collapse Score30

## References

[1] Brodsky JB, Cohen E (2000). Video-assisted thoracoscopic surgery. Curr Opin Anaesthesiol.

[2] Miyaji K, Ka K, Okamoto H, Takasaki T, Ohara K, Yoshimura H (2004). One-lung ventilation for video-assisted thoracoscopic interruption of patent ductus arteriosus. Surg Today.

[3] Nakayama M, Murray PA (1999). Ketamine preserves and propofol potentiates hypoxic pulmonary vasoconstriction compared with the conscious state in chronically instrumented dogs. Anesthesiology.

[4] Domino KB, Borowec L, Alexander CM, Williams JJ, Chen L, Marshall C (1986). Influence of isoflurane on hypoxic pulmonary vasoconstriction in dogs. Anesthesiology.

[5] Loer SA, Scheeren Thomas WL, Jorg T (1995). Desflurane inhibits hypoxic vasoconstriction in isolated rabbit lungs. Anesthesiology.

[6] Benumof JL (1986). Isoflurane anesthesia and arterial oxygenation during one-lung ventilation. Anesthesiology.

[7] Pruszkowski O, Dalibon N, Moutafis M, Jugan E, Law-Koune JD, Laloë PA (2007). Effects of propofol vs sevoflurane on arterial oxygenation during one-lung ventilation. Br J Anaesth.

[8] Beck DH, Doepfmer UR, Sinemus C, Bloch A, Schenk MR, Kox WJ (2001). Effects of sevoflurane and propofol on pulmonary shunt fraction during one-lung ventilation for thoracic surgery. Br J Anaesth.

[9] Reid CW, Slinger PD, Lenis S (1996). A comparison of the effects of propofol-alfentanil versus isoflurane anesthesia on arterial oxygenation during one-lung ventilation. J Cardiothorac Vasc Anesth.

[10] Sugasawa Y, Yamaguchi K, Kumakura S, Murakami T, Suzuki K, Nagaoka I (2012). Effects of sevoflurane and propofol on pulmonary inflammatory responses during lung resection. J Anesth.

[11] Voigtsberger S, Lachmann RA, Leutert AC, Schläpfer M, Booy C, Reyes L (2009). Sevoflurane ameliorates gas exchange and attenuates lung damage in experimental lipopolysaccharide-induced lung injury. Anesthesiology.

[12] Jin Y, Zhao X, Li H, Wang Z, Wang D (2013). Effects of sevoflurane and propofol on the inflammatory response and pulmonary function of perioperative patients with one-lung ventilation. Exp Ther Med.

[13] Sun B, Wang J, Bo L, Zang Y, Gu H, Li J (2015). Effects of volatile vs. propofol-based intravenous anesthetics on the alveolar inflammatory responses to one-lung ventilation: a meta-analysis of randomized controlled trials. J Anesth.

[14] Wigmore TJ, Mohammed K, Jhanji S (2016). Long-term Survival for Patients Undergoing Volatile versus IV Anesthesia for Cancer Surgery: A Retrospective Analysis. Anesthesiology.

[15] Wiklund CU, Lim S, Lindsten U, Lindahl SG (1999). Relaxation by sevoflurane, desflurane and halothane in the isolated guinea-pig trachea via inhibition of cholinergic neurotransmission. Br J Anaesth.

[16] Jiang G, Yang F, Li X, Liu J, Li J, Zhao H (2011). Video-assisted thoracoscopic surgery is more favorable than thoracotomy for administration of adjuvant chemotherapy after lobectomy for non-small cell lung cancer. World J Surg Oncol.

[17] Yoshimura T, Ueda K, Kakinuma A, Sawai J, Nakata Y (2014). Bronchial blocker lung collapse technique: nitrous oxide for facilitating lung collapse during one-lung ventilation with a bronchial blocker. Anesth Analg.

[18] Laster MJ, Fang Z, Eger EI (1994). Specific gravities of desflurane, enflurane, halothane, isoflurane, and sevoflurane. Anesth Analg.

[19] Joyce CJ, Williams AB (1985). Kinetics of absorption atelectasis during anesthesia: a mathematical model. J Appl Physiol.

[20] Lesitsky MA, Davis S, Murray PA (1998). Preservation of hypoxic pulmonary vasoconstriction during sevoflurane and desflurane anesthesia compared to the conscious state in chronically instrumented dogs. Anesthesiology.

[21] Ko R, McRae K, Darling G, Waddell TK, McGlade D, Cheung K (2009). The use of air in the inspired gas mixture during two-lung ventilation delays lung collapse during one-lung ventilation. Anesth Analg.

[22] Pfitzner J, Peacock MJ, Pfitzner L (2001). Speed of collapse of the non-ventilated lung during one-lung anaesthesia: the effects of the use of nitrous oxide in sheep. Anaesthesia.

[23] Joyce CJ, Baker AB, Parkinson R, Zacharias M (1996). Nitrous oxide and the rate of gas uptake from an unventilated lung in dogs. Br J Anaesth.

[24] Talbot NP, Balanos GM, Dorrington KL, Robbins PA (1985). Two temporal components within the human pulmonary vascular response to approximately 2 h of isocapnic hypoxia. J Appl Physiol.

[25] De Conno E, Steurer MP, Wittlinger M, Zalunardo MP, Weder W, Schneiter D (2009). Anesthetic-induced improvement of the inflammatory response to one-lung ventilation. Anesthesiology.

[26] Schilling T, Kozian A, Kretzschmar M, Huth C, Welte T, Bühling F (2007). Effects of propofol and desflurane anaesthesia on the alveolar inflammatory response to one-lung ventilation. Br J Anaesth.

[27] Schilling T, Kozian A, Senturk M, Huth C, Reinhold A, Hedenstierna G (2011). Effects of volatile and intravenous anesthesia on the alveolar and systemic inflammatory response in thoracic surgical patients. Anesthesiology.

[28] Cho YJ, Kim TK, Hong DM, Seo J-H, Bahk J-H, Jeon Y (2017). Effect of desflurane-remifentanil vs. propofol-remifentanil anesthesia on arterial oxygenation during one-lung ventilation for thoracoscopic surgery: a prospective randomised trial. BMC Anesthesiol.

[29] de la Gala F, Piñeiro P, Reyes A, Vara E, Olmedilla L, Cruz P (2017). Postoperative pulmonary complications, pulmonary and systemic inflammatory responses after lung resection surgery with prolonged one-lung ventilation. Randomized controlled trial comparing intravenous and inhalational anaesthesia. Br J Anaesth.

[30] Yuki K, Eckenhoff RG (2016). Mechanisms of the immunological effects of volatile anesthetics: a review. Anesth Analg.

[31] Yuki K, Hou L, Shibamura-Fujiogi M, Koutsogiannaki S, Soriano SG (2021). Mechanistic consideration of the effect of perioperative volatile anesthetics on phagocytes. Clin Immunol.

[32] Yap A, Lopez-Olivo MA, Dubowitz J, Hiller J, Riedel B (2019). Global Onco-Anesthesia Research Collaboration Group. Anesthetic technique and cancer outcomes: a meta-analysis of total intravenous versus volatile anesthesia. Can J Anaesth.

[33] Beck-Schimmer B, Bonvini JM, Braun J, Seeberger M, Neff TA, Risch TJ (2016). Which anesthesia regimen is best to reduce morbidity and mortality in lung surgery?: A Multicenter Randomized Controlled Trial. Anesthesiology.

